# Abundance and seasonal activity of *Haemaphysalis concinna* (Acari: Ixodidae) at the border between China and Russia in Northern Inner Mongolia, China

**DOI:** 10.1186/s13071-015-1291-6

**Published:** 2016-01-04

**Authors:** Hao Meng, Shiqi Xu, Zhijun Yu, Ningxin Li, Rongrong Wang, Xiaohe Gao, Xiaolong Yang, Jingze Liu

**Affiliations:** Key Laboratory of Animal Physiology, Biochemistry and Molecular Biology of Hebei Province, College of Life Sciences, Hebei Normal University, Shijiazhuang, 050024 China; Department of Pathogenic Biology, Hebei Medical University, Shijiazhuang, 050017 China; College of Basic Medicine, Chengde Medical University, Chengde, 067000 China

**Keywords:** *Haemaphysalis concinna*, Relative density, Seasonal activity, China-Russia border

## Abstract

**Background:**

*Haemaphysalis concinna*, a three-host tick vector of several pathogens, poses a high risk to the health of humans and livestock. However, knowledge of the seasonal activities, relative density and other ecological characteristics of this tick is quite limited and fragmentary. This knowledge gap represents a bottleneck in our understanding of the health risks associated with tick-borne pathogens.

**Methods:**

We conducted a two-year study from April 2012 to March 2014 in Northern Inner Mongolia situated on the China-Russia border, China, to investigate the seasonal activities and relative density of the three developmental stages of *H. concinna*. During the study period, feeding ticks were removed weekly from domestic sheep and their attachment sites were recorded. Questing ticks were collected weekly from five habitats (broadleaf forest, coniferous forest, shrubs, grassland and mixed coniferous forest) using the flagging-dragging method of capture. Rodents were captured and examined on two consecutive nights each week from June to September in 2012.

**Results:**

*H. concinna* ticks were found mainly in shrubs and grasslands habitats. Adults were encountered from February to October with the major peak occurring in June. Larvae, which were observed mainly from late April to late September, reached peak numbers in late July. Nymphs were observed mainly from March to October, and their numbers peaked in early July. *H. concinna* adults and nymphs were found attached to sheep and their most favored sites of attachment were the face and ears. *H. concinna* larvae were found on two rodent species, *Apodemus peninsulae* and *Eutamias sibiricus*.

**Conclusion:**

The relative density and seasonal activities of *H. concinna* have been systematically reported for Northern Inner Mongolia, China. The information about the hosts infested by *H. concinna* and its preferred attachment sites on sheep will help efforts to control this tick and the tick-borne diseases carried by it.

## Background

Ticks are arthropods with great medical value and veterinary importance [1, 2]. However, as the vectors of viruses, bacteria and protozoa, they also represent an important hazard to human and animal health world-wide [3, 4]. Tick-borne diseases are ranked highly in terms of their impact on the livelihood of resource-poor farming communities in developing countries [5]. In recent years, tick-borne diseases have occurred in almost all provinces of China and in the Russian Far East [6–8]. Therefore, studying tick ecology is a pivotal step towards obtaining a better understanding of the risk posed to animal and human populations by these arthropods [9].

*Haemaphysalis concinna* Koch is a three-host tick of significant importance because it serves as a vector of several pathogens of humans and livestock. It has been reported that *H. concinna* may carry Lyme borreliosis spirochetes [10], Far-Eastern subtype of tick-borne encephalitis virus [11], *Coxiella burnetii* [12], *Rickettsia sibirica* [1] and Crimean-Congo hemorrhagic fever virus [13]. *H. concinna* is distributed widely in China [14], Russia and Poland, as well as some parts of temperate Eurasia [15, 16]. In north China, this species was reported to attack domestic animals and humans, with heavy infestations occurring in summer [17].

A previous study indicated that *H. concinna* is abundant from spring to summer, but absent during winter in Cangxi County, Sichuan Province, China [18]. However, knowledge of the seasonal activities, relative density and other ecological characters of this tick species is still limited and fragmentary. The lack of detailed knowledge of tick ecology represents a bottleneck to better understanding of the risk of tick-borne pathogen introduction in this region of China [19]. Hence, the present study aimed to investigate the relative density and seasonal feeding and questing activities of *H. concinna* along the China-Russia border in Northern Inner Mongolia, China. The work may help predict the seasonality of tick-borne diseases and has potential to support the development of strategies for effective tick control.

## Methods

### Study site

**Fig. 1:**
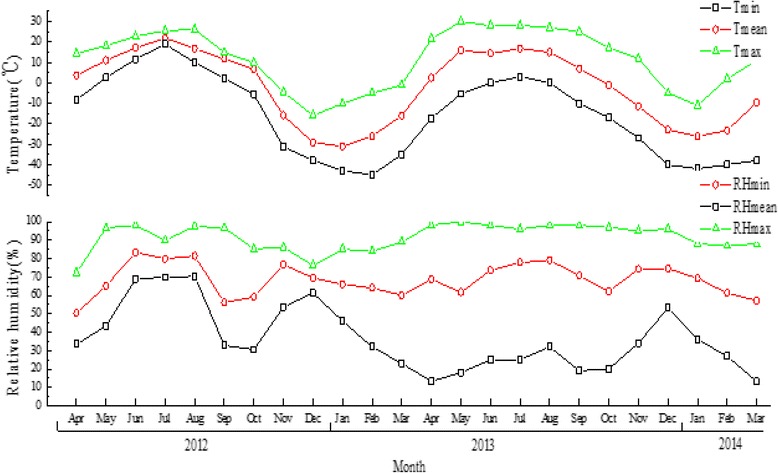
Monthly minimum (T_min_), maximum (T_max_) and mean (T_mean_) air temperature (°C) and minimum (RH_min_), maximum (RH_max_) and mean (RH_mean_) relative humidity (%) from April 2012 to March 2014, in Inner Mongolia, northeast China

This study was carried out from April 2012 to March 2014 in the Eerguna National Natural Reserve Area (120°00′26″to 120°58′02″E, 51°29′25″ to 52°06′00″N), which is situated on the China-Russia border in Northern Inner Mongolia, China. This area links China to the Russian Far East and has a cold temperate continental climate. The lowest temperature in winter and the highest temperature in summer were −45 °C and 33 °C, respectively. Monthly minimum, maximum and mean air temperatures and relative humidity from April 2012 to March 2014 were obtained by hygrothermographs (Qingsheng Electronic Technology Ltd., China) positioned about 50 cm above the ground (Fig. 1).

### Ticks on sheep

Ten randomly selected domestic sheep were tagged and allowed to graze in a natural pasture. Tick collections were carried out weekly from April 2012 to March 2014. Ticks were collected between 8:00 and 10:00 while the sheep were restricted. The ten sheep selected for this investigation were not treated with acaricides during the study period.

To determine the preferred attachment sites of the ticks, the body surface of each sheep was divided into seven areas: face, ears, neck, horn bases, back, belly and legs. All attached ticks were removed with a pair of forceps. The ticks collected were identified for species using a stereoscopic microscope [14] and counted after being brought back to the laboratory.

### Ticks on rodents

To investigate the presence of ticks on small mammals, rodents were captured for two consecutive nights each week under natural conditions from June to September in 2012. Two hundred live traps with the measure of 30 × 15 × 10 cm (Sichuan Shujile Trading Co., Chengdu, Sichuan, China) were placed along five lines covering an area of about 10000 m^2^. The traps, set up at around 18:00 h, were checked the following morning at around 07:00 h, as described previously [20]. Trapped rodents were anaesthetized by injection with 2 % pelltobarbitalum natricum (Tianjin Yongda Chemical Reagent Co., Tianjin, China) at a dose of 0.2 ml/100 g per bodyweight and identified for species according to a previous report [21]. The rodents were checked carefully and all the attached ticks were removed with forceps. Ticks at all three developmental stages were collected and identified [14]. The rodents were released back to the wild in their field of capture after tick removal. The prevalence of infestation was defined as the number of infested rodents divided by the total number of the host species examined, and the mean intensity represents the total number of ticks collected from each rodent species divided by the total number of infested hosts [20].

### Questing ticks

From March to October in 2012 and 2013, the seasonal activities of the questing stages of *H. concinna* were monitored. Five 1000 m^2^ plots were chosen from five different habitat types representing broadleaf forest (120°04′08″E, 51°35′06″N), coniferous forest (120°03′12″E, 51°37′38″N), shrubs (120°03′20″E, 51°37′01″N), grassland (120°03′09″E, 51°35′06″N) and mixed coniferous forest (120°04′02″E, 51°36′07″N), receptively. We used a 1.2 m × 1 m flannel cloth to observe the ticks, which was pulled weekly over the vegetation in the plots [2, 22]. Relative density was defined as the number of ticks collected in a 1000 m^2^ plot. Sampling was performed between 09:00 and 11:00 h at each site, and the flannel cloth was checked every 20 m. All the ticks attached to the flannel cloth were removed using tweezers and kept in vials for subsequent counting and identification.

### Statistical analysis

Statistical analyses were performed using STATISTICA Version 6.0 (StatSoft, Tulsa, OK, USA). Analysis of variance (ANOVA) and the correlation coefficient (r) were adopted to compare the collections in different biotopes and the relative density between the feeding and the questing ticks during the research period. The prevalence and mean intensity of infestation of different rodents by *H. concinna* larvae were analyzed using chi-squared analysis with the Bonferroni adjustment and the Kruskal–Wallis ANOVA test. Origin 7.0 (Microcal, Northampton, MA, USA) and Microsoft Excel were used to draw the figures and manage the data, respectively.

## Results

### Seasonality and relative density of *H. concinna* on sheep

**Fig. 2:**
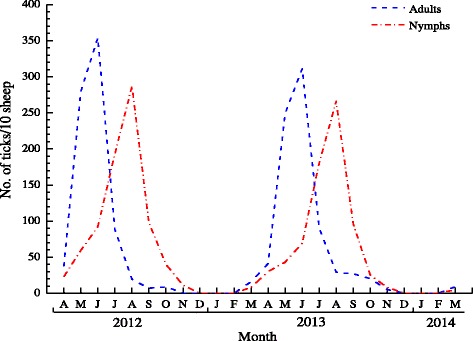
Intensity of infestation of sheep with *H. concinna* from April 2012 to March 2014. Letter A, M, J, J, A, S, O, N, D represent April, May, June, July, August, September, October, November and December, respectively

A total of 3124 ticks, including 1590 adults and 1534 nymphs, were collected from sheep during the two-year study period. Adults were found from February to October with a major peak in June. Infestation with nymphs started in February, reached its peak in August and continued until November in both study years (Fig. 2). No adults or nymphs were found on sheep in the winter months. No larvae were found on sheep during this study.

### Attachment sites on sheep

The face and ears were found to be the preferred attachment sites for *H. concinna*. 59 % (1843 ticks) of the total number of ticks were found on the face and 33 % (1031 ticks) of ticks were found on the ears. Only 8 % (250 ticks) of the ticks were found on the other body parts of the sheep.

### Seasonality of rodent infestation by larvae

**Fig. 3:**
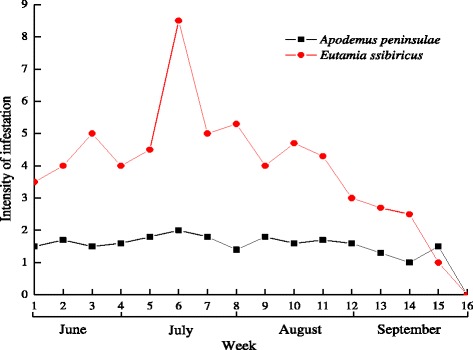
Intensity of infestation of rodents with *H. concinna* larvae from June 2012 to September 2014

A total of 258 rodents, including 198 *Apodemus peninsulae* and 60 *Eutamias sibiricus*, were captured between June and September of 2012. A total of 415 *H. concinna* larvae were collected from the rodents captured during the surveys and they appeared from June to September with an activity peak in mid-July. 146 *A. peninsulae* and 43 *E. sibiricus* were infested by larvae of *H. concinna*, respectively. The statistical analysis showed no significant differences in the prevalence (*P* > 0.05) and significant differences in mean intensity of infestation by tick larvae between *A. peninsulae* and *E. sibiricus* (*P* < 0.05). Prevalence and mean intensity of infestation by tick larvae on rodents were presented in Table 1. A few nymphs (<10) and no adults were collected from the capture rodents. The seasonality of infestation of rodents by larvae is shown in Fig. 3.

**Table 1:** Prevalence and mean intensity of infestation of rodents by *H. concinna* larvae

Host			Larvae		
Species	No. of total	No. of infested	Prevalence	n	Mean intensity
*Apodemus peninsulae*	198	146	73.7	235	1.6^a^
*Eutamias sibiricus*	60	43	71.7	180	4.2^b^
Total	258	189	-	415	-

Different superscript letters indicate statistical differences between data (*P <* 0*.*05)

### Seasonal changes in relative density of questing ticks

**Fig. 4:**
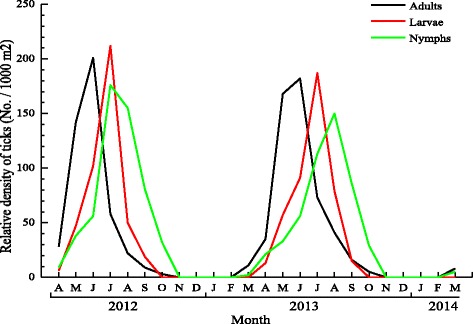
Seasonal activity of questing *H. concinna* in shrubs and grassland from April 2012 to March 2014. Letter A, M, J, J, A, S, O, N, D represent April, May, June, July, August, September, October, November and December, respectively

In total, 2916 questing *H. concinna*, including 996 adults, 878 larvae, and 1042 nymphs were collected from shrubs and grassland habitats. However, only six ticks were collected from broadleaf and mixed coniferous forests. The activities of the three developmental stages of *H. concinna* in shrubs showed distinct seasonal patterns. In both years, the adults were observed mainly from March to September and peaked in early June. Larvae were observed mainly from late April to late September and they displayed an activity peak in late July. Nymphs were observed mainly from March to October and peak observation was in August (Fig. 4). In contrast, no questing ticks were collected in the winter months (Fig. 4).

## Discussion

The border region between China and the Far-Eastern region of Russia has a cold temperate continental climate with an annual mean temperature of −3 °C (ranged from −46 to 34 °C), an annual mean rainfall of 400 mm (ranged from 414 to 528 mm), and five different habitat types including broadleaf forest, coniferous forest, shrubs, grassland and mixed coniferous. Because of the long cold weather in winter and the relatively short hot weather in summer, there was much overlap between the activities of three different questing stages of *H. concinna* in the hottest months (Fig. 4). The seasonal activities of *H. concinna* in temperate Europe overlap with the three different questing stages during the months of June, July and August [15]. Similar activity patterns of different developmental stages were also reported in *H. longicornis* [22] and *H. punctata* [23].

The distribution patterns of ticks in one location can vary because of fluctuations in the relative density of host animals or human interventions [24]. Correlations between tick population density and host density have been reported in numerous investigations conducted globally [25, 26]. In the present study, significantly high numbers of ticks were found in grassland and shrubs, unlike the three other habitat types. Different host density is likely to be the major factor contributing to the inter-site differences we observed. Sheep graze mainly in grassland and shrubs and rarely enter other areas. Grassland and shrubs may act as shelter for rodents. These areas probably provide plenty of hosts for ticks, thereby improving their survival rates. If newly molted ticks were put on a host immediately, *H. concinna* can complete one generation per year in the field [27]. This finding is similar to previous results illustrating that host activity plays an important role in where ticks are distributed in the environment [28–30].

Previous studies indicate that *H. concinna* adult-stage ticks feed mainly on large-size animals, and occasionally attack humans. The most important hosts for immature stages of *H. concinna* are small- to medium-sized mammals [15]. Some surveys have been carried out to investigate the occurrence of ticks infesting dogs [31, 32], and one survey reported that the infestation intensity ranged from 1 to 78 ticks per dog [31]. In the present study, sheep and rodents were chosen as the hosts for ticks and the intensity of infestation is shown in Figs. 2 and 3. Wild ruminants usually carry ticks of all life stages in the field. Roe deer and goats were found to be the important hosts for the larvae and nymphs of *H. concinna* in South Hungary [33]. However, data on such hosts of *H. concinna* are limited in China because of the difficulty in capturing them*.* Nevertheless, only a few (*n* < 10) nymphs were collected from the rodents captured in our study area, although many more nymphs were collected from sheep. Additionally, larvae were only detected on rodents, and adults only captured from sheep. Our statistical analysis showed that the infestation prevalence in *A. peninsulae* did not differ significantly from that of *E. sibiricus* (*P* > 0.05), but the mean intensity of infestation did differ significantly between the two host species (*P* < 0.05). Larvae were not found on large-sized mammals such as sheep, but they were present on small-sized rodents. This study further revealed the host selection of *H. concinna* under natural conditions at the border between China and Russia in Northern Inner Mongolia, China.

A large number of studies on tick infestation have been conducted, in which, the whole host body was divided into a small number of areas [34, 35]. In our study, we divided the sheep body into the face, ears, neck, horn bases, and other body parts. The results showed that 59 and 33 % of the total number of *H. concinna* ticks were distributed on the face and ears, respectively. In contrast, only 8 % of the total ticks collected were found on the other body parts of the sheep. Our result is similar to previous findings for other tick species, including *H. bispinosa*, *H. intermedia* [36], *H. longicornis* [22], *Ixodes ricinus* and *Dermacentor reticulatus* [35]. The face and ears of sheep are at the front while walking through the vegetation and more importantly, the skin of these areas is thin. The finding of major attachment sites of ticks on sheep would allow the targeted application of pesticides to control ticks on sheep.

The adult and nymphal populations of *H. concinna* could be controlled by acaricide application on sheep from late April to June. The information provided by our study concerning the preferred attachment sites of ticks could help to improve the effectiveness of acaricides by targeting both the face and ears of sheep susceptible to tick infestation.

## Conclusions

Our study has provided data on the relative density and seasonal activities of *H. concinna* on the China-Russia border in Northern Inner Mongolia, China. The relative density and seasonal activities of *H. concinna* were studied in questing ticks and ticks infesting sheep and rodents. The face and ears of sheep were found to be the preferred attachment sites for *H. concinna*. These results should help raise awareness in people living in this region of China about the health risks associated with tick bites, and that ticks are more likely to be encountered in the natural environment from late April to June.

### Ethical approval

Experiments involving animals were approved by the Animal Ethics Committee of Hebei Normal University (Protocol number: IACUC-127001).
